# Impact of acupuncture on muscle electromyography poststroke: A narrative review

**DOI:** 10.1097/MD.0000000000043213

**Published:** 2025-08-01

**Authors:** Jiahui Liu, Yuanliang Chen, Sike Peng, Hongmei Cheng, Yu He, Hongkun Ye, Chongrui Li, Yisen Wu, Jingtian Wang, Xinyin Xu

**Affiliations:** a College of Acupuncture and Orthopedics, Hubei University of Chinese Medicine, Wuhan, Hubei, China; b Hubei Provincial Hospital of Traditional Chinese Medicine, Wuhan, China; c Hubei Shizhen Laboratory, Wuhan, China; d The First Clinical College, Hubei University of Chinese Medicine, Wuhan, China.

**Keywords:** acupuncture, alternative medicine, complementary medicine, electromyography, mechanisms, review, stroke

## Abstract

We reviewed the effects of acupuncture on muscle electrophysiology in stroke patients, with an emphasis on the factors influencing these effects and the underlying mechanisms. A comprehensive search of Chinese and English literature was conducted using databases including China National Knowledge Infrastructure, PubMed, and Web of Science. The search covered studies published between 1990 and 2023, using MeSH terms and keywords such as “electromyography,” “acupuncture,” “stroke,” and “mechanisms.” After excluding duplicates, irrelevant articles, phase I clinical trials, meta-analyses, reviews, case reports, and case series, 32 original studies and clinical trials with well-defined methodologies and results were selected from an initial pool of 1587 publications. Snowballing techniques were also used to identify additional relevant literature. Acupuncture was found to exert a bidirectional regulatory effect on electromyographic activity, enhancing or inhibiting it depending on variables such as stimulation method, frequency, duration, timing, and acupoint selection. The variability of outcomes highlights the complexity of electromyographic modulation, which likely involves integrated physiological responses including neural pathways, neurotransmitters, related proteins, receptors, and other biomolecules. Future research should focus on elucidating these mechanisms to optimize the application of acupuncture in poststroke rehabilitation.

## 1. Introduction

Clinical trials have verified that acupuncture is a beneficial treatment for stroke.^[1]^ The epidemiological characteristics of this study are described as follows.^[2]^ Firstly, the study was conducted only in China, providing the majority of the data. The age distribution ranged from 30 to 65 years, with most cases concentrated in the 40 to 64 age group. In terms of gender, the prevalence of stroke was higher in men than in women, and the incidence among men showed a year-on-year increasing trend, whereas the trend among women remained relatively stable. Geographically, the distribution of stroke incidence, prevalence, and mortality in China followed a distinct pattern: higher in the North, lower in the South, and particularly pronounced in the central regions. Lastly, common comorbidities among the study population included hypertension, diabetes, dyslipidemia, and heart disease. Tests of electromyographic activity, such as surface electromyography and intramuscular electromyography, are accessible and noninvasive or minimally invasive. These tests provide objective bioelectrical indices for evaluating motor function in stroke hemiplegia, offering a more precise and quantitative assessment of patients’ muscle status and rehabilitation stage compared to traditional scoring scales.

This article examines the impact of acupuncture on the regulation of EMG in stroke patients and the variables influencing this regulation. It also provides an overview of how acupuncture modulates the electromyography activity in stroke patients.

## 2. Methods

### 2.1. Selection criteria and search strategy

**Figure 1.**
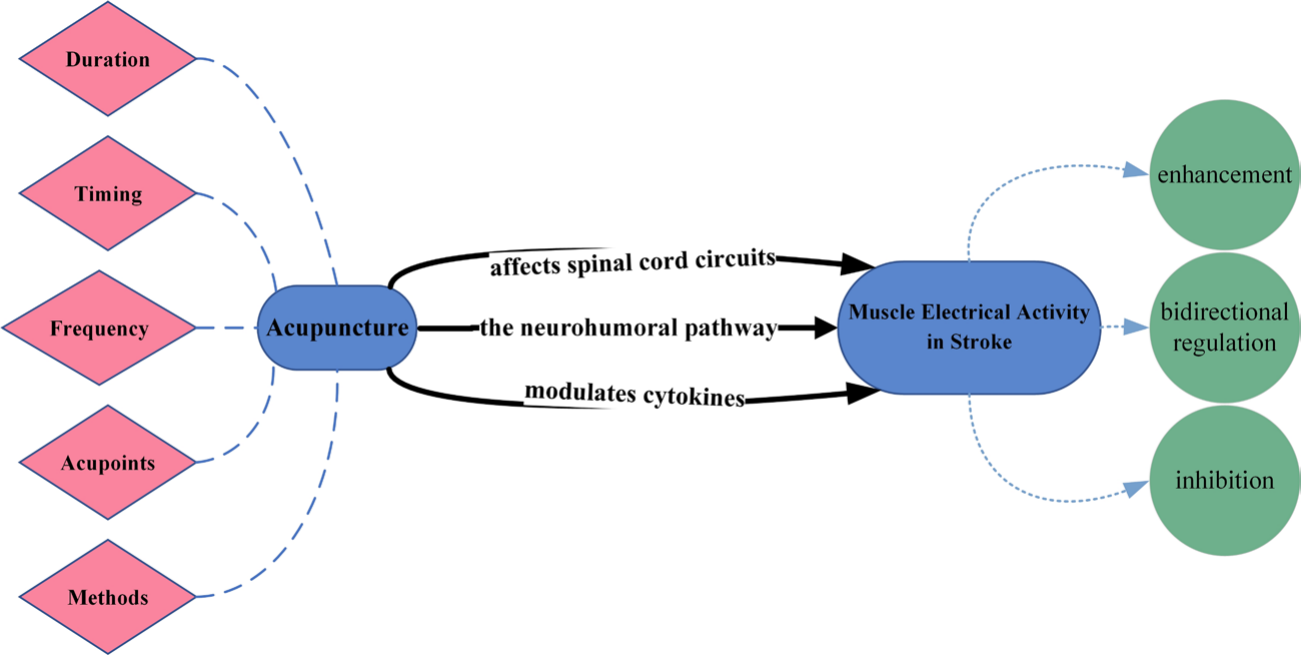
Diagram of article relationships.

To conduct this review, we performed a comprehensive literature search in both Chinese and English databases. Specifically, we searched the China National Knowledge Infrastructure for Chinese literature and PubMed and Web of Science for English literature. Studies classified as phase I clinical trials, meta-analyses, review articles, case reports, and case series were excluded. After removing duplicates, nonrelevant studies, and review articles, a total of 32 studies were selected from an initial pool of 1587 publications. These studies focused on acupuncture’s effect on muscle electrophysiology in stroke patients, as summarized in Figure 1. Our search strategy incorporated both MeSH terms and relevant keywords, including “electromyography,” “acupuncture,” “stroke,” and “mechanisms” (Table 1). We included studies published between 1990 and 2023 and prioritized original research and clinical trials with well-defined methodologies and results. To further supplement our selection, we employed the snowballing technique to identify additional relevant references. Moreover, we explored potential strategies to enhance the effectiveness of acupuncture in stroke rehabilitation.

**Table 1:** The search strategy summary.

Items	Specification
Date of search	July 3, 2022 to June 27, 2024
Databases and other sources searched	CNKI, PubMed, Web of Science
Search terms used	“Acupuncture” AND “Stroke” [MeSH] │ “Acupuncture” AND “Electromyography” [MeSH] │ “Acupuncture” AND “EMG” AND “Stroke” [MeSH] │ “Acupuncture” AND “sEMG” AND “Stroke” [MeSH] │ “Acupuncture” AND “Electromyographic activity” AND “Stroke” [MeSH] │ “Acupuncture” AND “Surface electromyography” AND “Stroke” [MeSH] │ “Electromyography” [MeSH] AND “H-reflex” │ “Electromyography” [MeSH] AND “F-wave” │ (“Acupuncture” OR “H-reflex” OR “F-wave”) AND “Stroke” [MeSH] AND “Mechanisms” [MeSH] │ (“Acupuncture” OR “H/M value”) AND “Stroke” [MeSH] AND “Mechanisms” [MeSH] │ “Acupuncture” AND “Stroke” [MeSH] AND “Electromyography” [MeSH] AND “Neuron” │ “Acupuncture” AND “Poststroke spasticity” AND “Electromyography” [MeSH] AND “Neuron”
Timeframe	1990–2023
Inclusion and exclusion criteria	Inclusion criteria: Research articles and clinical trials in Chinese and English focusing on themes such as acupuncture, electromyographic activity, and mechanisms. Exclusion criteria: Studies deemed to have low reliability based on methodological quality and scientific rigor.
Selection process	The included literature was selected by Jiahui Liu and reviewed by all authors.
Any additional considerations, if applicable	Some papers were identified by reviewing reference lists of relevant publications.

CNKI = China National Knowledge Infrastructure, EMG = electromyography, sEMG = surface electromyography.

Since this is a narrative review, ethical approval was not required.

## 3. Discussion

### 3.1. Modulation of electromyography activity in stroke by acupuncture

Acupuncture has a variable modulatory effect on the electromyography activity in stroke patients. It enhances activity during the flaccid phase, inhibits activity during the spastic phase, and provides bidirectional regulation during the recovery phase.

#### 3.1.1. Enhancement during the flaccid phase

In the flaccid phase, characterized by low muscle tone and decreased tendon reflexes, strong stimulation acupuncture can increase EMG, improving muscle tone and strength. Liu et al^[3]^ investigated the clinical efficacy of acupuncture in the treatment of poststroke dysphagia and found that the maximum amplitude of surface electromyography (sEMG) in the suprahyoid muscle group was significantly higher in the acupuncture group than in the control group receiving conventional rehabilitation training. These results indicate that acupuncture enhances EMG and suggests that it can effectively improve the contractile ability of swallowing-related muscles. Similarly, Zhang et al^[4]^ reported that the standardized average electromyographic value, peak value, and integrated electromyographic value (IEMG) in patients with poststroke dysphagia were significantly higher in the acupuncture group than in the control group undergoing conventional swallowing training. These findings suggest that acupuncture increases the intensity of electromyographic signals, demonstrating its excitatory effect on EMG.

#### 3.1.2. Inhibition during the spastic phase

Acupuncture also effectively reduces muscle spasticity in stroke patients. Zhao et al^[5]^ reported that acupuncture significantly lowered EMG values in the upper and lower limbs, improving spasticity in stroke patients. Xin et al^[6]^ noted a substantial reduction in sEMG signals during swallowing movements before and after acupuncture treatment. Deng et al^[7]^ found that average electromyographic values decreased from the first to the third day after acupuncture, indicating reduced muscle spasticity in stroke patients.

#### 3.1.3. Bidirectional regulation during the recovery phase

During the recovery phase, acupuncture provides bidirectional regulation, enhancing antagonist muscle function and reducing agonist muscle tone in stroke patients. Wu et al^[8]^ demonstrated that acupuncture and extracorporeal shock wave therapy had a bidirectional effect on the surface electromyography of the rectus femoris and biceps femoris, improving muscle coordination in stroke patients. Wang et al^[9]^ found that electroacupuncture at various frequencies decreased IEMG in the rectus femoris and gastrocnemius muscles while increasing IEMG in the anterior tibial muscles. This balancing effect contributes to improved motor function in stroke patients. Huang et al^[10]^ investigated the impact of combined stabbing and cupping therapy on upper limb spasticity, finding that it effectively improved elbow flexion spasm and motor function by balancing the IEMG of the biceps brachii and triceps brachii muscles in stroke patients. Wang et al^[11]^ compared the clinical efficacy of acupuncture combined with rehabilitation training versus rehabilitation training alone in the treatment of foot drop following stroke. Their study revealed that, compared to the control group receiving only conventional rehabilitation, the combined treatment group exhibited a more pronounced decrease in the IEMG and co-contraction ratio of the gastrocnemius muscle. Similarly, Wang et al^[12]^ examined the effects of acupuncture on lower limb spasticity in patients with hemorrhagic stroke. Their findings demonstrated that, in comparison with the control group receiving only standard treatment, the IEMG of spastic agonist muscles significantly decreased, while that of spastic antagonist muscles markedly increased.

Acupuncture’s bidirectional modulation of EMG helps restore normal function and balance in stroke patients, contributing to improved outcomes in various phases of recovery.

### 3.2. Research on variables influencing electromyography activity of stroke muscles adjusted by acupuncture

Acupuncture’s efficacy in regulating EMG in stroke patients depends on several parameters, including method, frequency, duration, timing, and acupoint selection.

#### 3.2.1. Impact of acupuncture duration

Hu et al^[13]^ found that different acupuncture retention times at the acupoints ST36, SP6, ST36, and LR3 significantly affected treatment outcomes. Specifically, retaining the needle for 30 minutes resulted in the most significant improvement in synergistic contraction rates. Similarly, Wang et al^[14]^ demonstrated that 30-minute needle retention was more effective in reducing IEMG and the H/M ratio in patients with poststroke lower limb spasms compared to immediate removal or 60-minute retention.

#### 3.2.2. Impact of acupuncture timing

Fan et al^[15]^ reported that acupuncture performed at specific times, coordinated with the heavenly stem and earthly branch, significantly reduced the H/M max index and improved muscle tone and daily activity performance in patients with poststroke spastic hemiparesis compared to conventional meridian acupoint acupuncture.

#### 3.2.3. Impact of acupuncture frequency

Wang et al^[16]^ investigated the effects of different electroacupuncture frequencies on stroke patients with lower limb spasticity. They found that 100 Hz electroacupuncture significantly increased IEMG in the sartorius and tibialis anterior muscles while reducing it in the gastrocnemius and rectus femoris muscles. In contrast, 2 Hz electroacupuncture had no significant effect on spasmodic muscles’ electromyography. Chen et al^[17]^ also observed that high-frequency acupuncture reduced root-mean-square more effectively than low-frequency acupuncture in treating hemiplegic elbow flexor spasms.

#### 3.2.4. Impact of acupuncture acupoints

Wang et al^[18]^ compared the effects of warm acupuncture and electroacupuncture on stroke patients with flaccid paralysis. Warm acupuncture was superior in improving IEMG, root-mean-square, and co-contraction ratio of the gastrocnemius and tibialis anterior muscles during ankle flexion and extension. He et al^[19]^ noted that combining head and body acupuncture significantly improved motor dysfunction, with higher IEMG scores for the triceps brachii and tibialis anterior muscles compared to control groups. These findings suggest that targeting specific acupoints can enhance muscle tone and motor function in stroke patients.

#### 3.2.5. Impact of acupuncture methods

Wang et al^[20]^ evaluated 2 electroacupuncture treatment modalities for poststroke unilateral upper limb spastic hemiplegia: acupuncture at spasmodic side meridian points and non-spasmodic side meridian points. The study showed that acupuncture on the non-spasmodic limb significantly inhibited aberrant activity of spinal cord motor neurons, as indicated by a lower H/M max ratio compared to the spastic side.

These findings underscore the importance of optimizing acupuncture parameters, including duration, timing, frequency, acupoint selection, and methods, to effectively regulate EMG and improve outcomes for stroke patients.

### 3.3. Research on how acupuncture affects electromyography activity in stroke

#### 3.3.1. Spinal cord circuits are impacted by acupuncture

Acupuncture modifies limb function in stroke patients through multiple levels, pathways, and targets, possibly mediated by the H-reflex and F-wave brain circuits. The H reflex involves the action potential produced by peripheral nerves, which travels to the spinal cord and then to the α motor neurons, resulting in electromyography activity. It provides objective electrophysiological markers and represents the excitability of α motor neurons.^[21]^ According to Shan et al,^[22]^ combined electroacupuncture significantly improved the H/M max ratio of radial, ulnar, and median nerves, indicating reduced motor neuron excitability. Jin et al^[23]^ found that the electroacupuncture treatment group’s H/M value at the GB34 acupuncture point was significantly lower in model rats with cerebral infarction, suggesting inhibition of spinal cord motor neuron excitability. The action potential first travels to the muscle upon nerve stimulation and then along the motor neuron of the spinal cord, generating a posterior emission potential transmitted downward, causing secondary muscle excitation.^[24,25]^ F-wave detection is crucial for reflecting motor neuron excitability and peripheral nerve conduction pathways.^[26,27]^ Studies show that poststroke patients with limb spasticity experience a significant reduction in F-wave amplitude and area, reducing muscle tone and improving spasticity.^[28]^

Thus, acupuncture’s regulation of neural circuits may decrease motor neuron excitability, modifying nerve impulse generation and conduction, partially explaining its modulation of EMG in stroke patients.

#### 3.3.2. The neurohumoral route by acupuncture

Central neurotransmitters and their receptors also influence acupuncture’s effect. Excitatory neurotransmitters include glutamate and aspartic acid, while inhibitory ones include γ-aminobutyric acid (GABA), glycine, serotonin, norepinephrine, and dopamine. Spastic states with altered EMG can arise from imbalances in these neurotransmitters. Zhang et al^[29]^ found that acupuncture treatment for poststroke spasticity in rats increased glutamate, aspartic acid, and reactive oxygen species (ROS) levels while decreasing GABA, glycine, serotonin, dopamine, and norepinephrine levels, reducing EMG and the H reflex stimulation threshold. These findings suggest that acupuncture enhances neurotransmitter content, providing neuroprotection and anti-spasm effects. Therefore, acupuncture may regulate EMG in stroke patients by adjusting the brain’s metabolic state through the neurohumoral pathway, decreasing excitatory amino acids, increasing inhibitory amino acids, and inhibiting abnormal excitation.

#### 3.3.3. Cytokines are modulated by acupuncture

Acupuncture can affect EMG by influencing growth hormones and inflammatory cytokines. Qi et al^[30]^ studied a rat model of spasmodic cerebral ischemia and found a significant decrease in C-reactive protein, interleukin-6, tumor necrosis factor-α, and nitric oxide synthase levels. Electrophysiological tracings showed reduced muscle tone, suggesting that acupuncture might inhibit inflammatory cytokine release post-brain injury, reducing immune damage and muscle spasms. Liu et al^[31]^ found that acupuncture combined with medication increased the expression of cerebral cortical growth factors like tropomyosin receptor kinase B and brain-derived neurotrophic factor in rats with poststroke limb spasms, improving neurological function and reducing spasticity. Studies indicate a positive correlation between EMG indicators and muscle strength and tone scores.^[32,33]^

**Figure 2.**
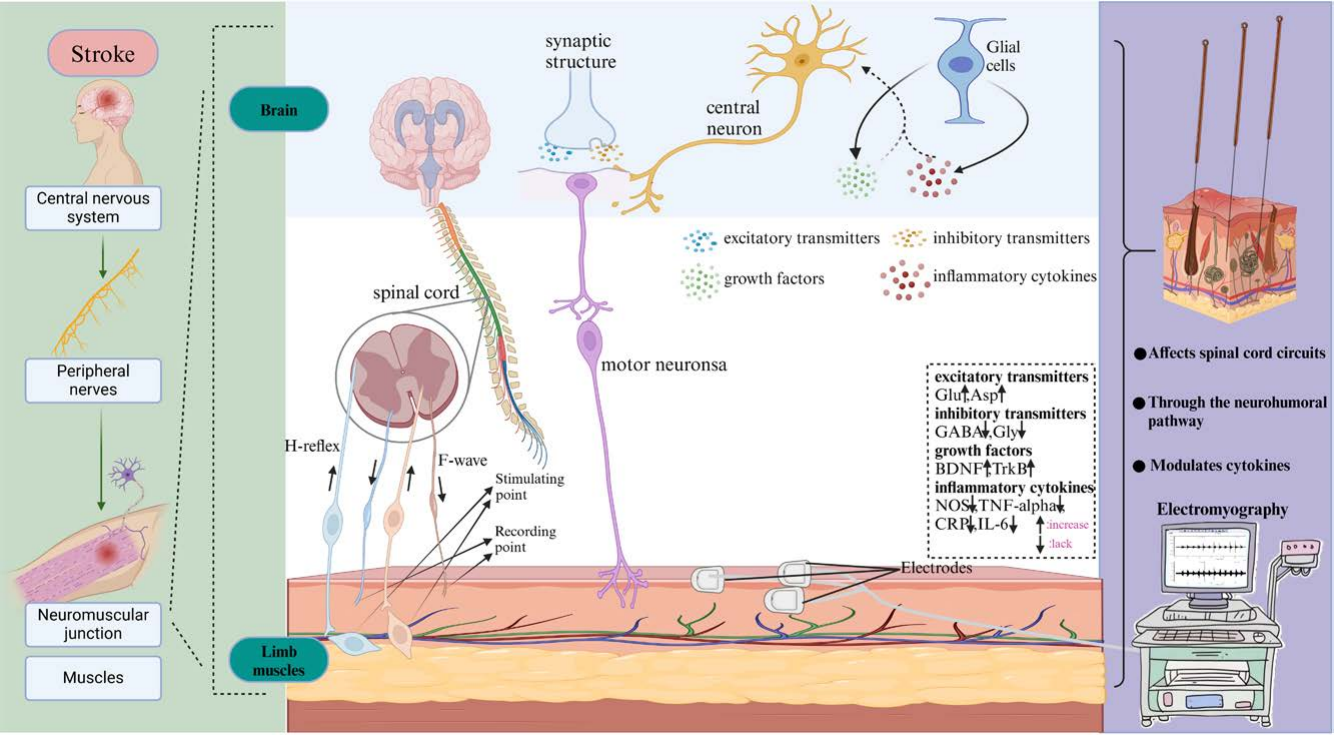
The mechanism by which stroke causes changes in electrical muscle activity after limb spasm, and how acupuncture effects electrical muscle activity through spinal cord circuits, neurohumoral pathways, and cytokines (created with BioRender.com).

The acupuncture effect on EMG regulation in stroke patients results from the comprehensive interaction of the nervous system, neurotransmitters, related proteins, and other biomolecules (Fig. 2). Further investigation is needed into the neurological pathways and associated proteins involved in this modulation.

## 4. Conclusions

EMG, reflecting the strength and function of the neuromuscular system, is the weak electrophysiological signals produced by muscle contractions. These signals are detected on the skin or in muscle tissue by electrodes and are frequently used to assess the health of individuals recovering from a stroke. Electromyography, known for its safety, ease of use, and noninvasive nature, has proven beneficial in various domains. However, its application in acupuncture faces technical challenges such as noise interference and action potential aliasing. Additionally, issues like individual variability, unclear clinical relevance of parameter changes, and the need for standardized validation present significant obstacles.

The muscle tone score, commonly used to evaluate spasticity, lacks objectivity, sensitivity, and real-time accuracy. Objective and quantitative EMG is less frequently applied, limiting the thorough assessment of muscle conditions.

Several factors influence how well acupuncture regulates EMG. Most research has focused on treatment-related factors like acupoint selection and acupuncture techniques, with little emphasis on patient-related aspects. Due to the reliance of neurotracing techniques and other research methods on invasive clinical trials, their application in human studies is subject to ethical constraints. Consequently, direct evidence on how acupuncture influences EMG via brain pathways remains limited. Future research should consider integrating noninvasive techniques, such as functional magnetic resonance imaging and near-infrared spectroscopy, to explore the regulatory mechanisms of acupuncture through brain network analysis; Moreover, studies on the combined mechanisms of acupuncture interventions remain insufficient, as most research focuses on isolated mechanisms while neglecting their synergistic interactions. This indicates that the multi-level and multi-dimensional mechanisms of acupuncture have yet to be systematically elucidated. Thus, future investigations should adopt a holistic regulatory framework to comprehensively elucidate acupuncture’s modulatory effects on multiple physiological systems, thereby establishing a robust theoretical foundation for its clinical applications. Existing research suggests that acupuncture may activate the neuro-endocrine-immune network by targeting key regulatory nodes, thereby facilitating interactions and coordination among the nervous, endocrine, and immune systems, ultimately influencing EMG. Further exploration of this mechanism will enhance our understanding of the neuromuscular regulatory effects of acupuncture and provide robust scientific support for its clinical applications.

To support clinical practice, it is crucial to identify and integrate the factors currently influencing the effectiveness of acupuncture. Developing comprehensive stroke treatment programs that move from single to multi-factor approaches is essential. Despite the increasing application of EMG examination, further large-sample and multi-center studies are needed to explore this technique. Such research is anticipated to broaden the application prospects of EMG examination in acupuncture.

## Acknowledgments

Figure 2 was created with BioRender.com. The authors wish to thank Dr Chengwei Fu of Hubei Provincial Hospital of Traditional Chinese Medicine for reading and revising the manuscript carefully.

## Author contributions

**Conceptualization:** Hongkun Ye, Chongrui Li.

**Formal analysis:** Sike Peng, Hongmei Cheng, Jingtian Wang.

**Funding acquisition:** Xinyin Xu.

**Methodology:** Yisen Wu.

**Supervision:** Yuanliang Chen.

**Validation:** Yu He.

**Writing – original draft:** Jiahui Liu.

**Writing – review & editing:** Jiahui Liu.
